# Impact of HACA on Immunomodulation and Treatment Toxicity Following ch14.18/CHO Long-Term Infusion with Interleukin-2: Results from a SIOPEN Phase 2 Trial

**DOI:** 10.3390/cancers10100387

**Published:** 2018-10-17

**Authors:** Nikolai Siebert, Sascha Troschke-Meurer, Madlen Marx, Maxi Zumpe, Karoline Ehlert, Juliet Gray, Alberto Garaventa, Carla Manzitti, Shifra Ash, Thomas Klingebiel, James Beck, Victoria Castel, Dominique Valteau-Couanet, Hans Loibner, Ruth Ladenstein, Holger N. Lode

**Affiliations:** 1 Department of Pediatric Oncology and Hematology, University Medicine Greifswald, 17475 Greifswald, Germany; nikolai.siebert@uni-greifswald.de (N.S.); sascha.troschke-meurer@uni-greifswald.de (S.T.-M.); madlen.marx@uni-greifswald.de (M.M.); maxi.zumpe@uni-greifswald.de (M.Z.); ehlertk68@uni-greifswald.de (K.E.); 2 Department of Paediatric Oncology, University Hospital Southampton, Southampton SO14 0YG, UK; Juliet.Gray@uhs.nhs.uk; 3 Oncology Unit, Istituto Giannina Gaslini, 16147 Genova, Italy; albertogaraventa@gaslini.org (A.G.); CarlaManzitti@gaslini.org (C.M.); 4 Pediatric Hemato-Oncology Division, Schneider Children Medical Center, Kaplan 14, Petach Tikva 4920235, Israel; Shifraa@clalit.org.il; 5 University Children’s Hospital, Goethe University Frankfurt, 60596 Frankfurt, Germany; Thomas.Klingebiel@kgu.de; 6 University Children’s Hospital, Friedrich Schiller University Jena, 07747 Jena, Germany; James.Beck@med.uni-jena.de; 7 Pediatric Hemato-Oncology Unit, University Hospital La Fe, 46026 Valencia, Spain; castel_vic@gva.es; 8 Pediatric and Adolescent Oncology, Gustave Roussy Université Paris-Sud, 94800 Villejuif, France; Dominique.VALTEAU@gustaveroussy.fr; 9 Advisor to Apeiron Biologics AG, 1030 Vienna, Austria; hans.loibner@apeiron-biologics.com; 10 St. Anna Children’s Hospital and Children’s Cancer Research Institute (CCRI), Department of Paediatrics, Medical University, Kinderspitalgasse 6, 1090 Vienna, Austria; ruth.ladenstein@ccri.at

**Keywords:** neuroblastoma, anti-GD_2_ immunotherapy, ch14.18/CHO, pain, long-term infusion, HACA, complement dependent cytotoxicity

## Abstract

GD_2_-directed immunotherapies improve survival of high-risk neuroblastoma (NB) patients (pts). Treatment with chimeric anti-GD_2_ antibodies (Ab), such as ch14.18, can induce development of human anti-chimeric Ab (HACA). Here, we report HACA effects on ch14.18/CHO pharmacokinetics, pharmacodynamics and pain intensity in pts treated by long-term infusion (LTI) of ch14.18/CHO combined with IL-2. 124 pts received up to 5 cycles of ch14.18/CHO 10 days (d) infusion (10 mg/m^2^/d; d8–18) combined with s.c. IL-2 (6 × 10^6^ IU/m^2^/d; d1–5, d8–12). HACA, treatment toxicity, ch14.18/CHO levels, Ab-dependent cellular- (ADCC) and complement-dependent cytotoxicity (CDC) were assessed using respective validated assays. HACA-negative pts showed a steadily decreased pain in cycle 1 (74% pts without morphine by d5 of LTI) with further decrease in subsequent cycles. Ch14.18/CHO peak concentrations of 11.26 ± 0.50 µg/mL found in cycle 1 were further elevated in subsequent cycles and resulted in robust GD_2_-specific CDC and ADCC. Development of HACA (21% of pts) resulted in strong reduction of ch14.18/CHO levels, abrogated CDC and ADCC. Surprisingly, no difference in pain toxicity between HACA-positive and -negative pts was found. In conclusion, ch14.18/CHO LTI combined with IL-2 results in strong activation of Ab effector functions. Importantly, HACA response abrogated CDC but did not affect pain intensity indicating CDC-independent pain induction.

## 1. Introduction

Disialoganglioside GD_2_ is a glycolipid overexpressed on neuroblastoma (NB) cells and clinical trials with anti-GD_2_ monoclonal Abs (mAb) have shown promising results [1]. The Children’s Oncology Group (COG) reported improved survival rates in NB pts treated with the human/mouse chimeric Ab ch14.18 produced in SP2/0 cells [2]. Given as a short-term infusion (4 × 25 mg/m^2^/d, 8–20 h) and in combination with cytokines, ch14.18 improved survival of high-risk NB pts [2]. In Europe, the production of ch14.18 in accordance with a Good Manufacturing Practice (GMP) was initiated by the International Society of Paediatric Oncology European Neuroblastoma Group (SIOPEN). In contrast to ch14.18 produced in SP2/0, the European industrial production of the Ab was performed in most commonly used mammalian cell line for industrial production of recombinant protein therapeutics, Chinese hamster ovary (CHO) cells [3]. A first clinical evaluation of the recloned Ab ch14.18/CHO was performed in a phase 1 bridging study based on short-term infusion (STI) of the Ab (5 × 20 mg/m^2^/d, 8 h infusion) [4]. In this study, pts treated with ch14.18/CHO showed similar characteristics compared to ch14.18 produced in SP2/0 cells including toxicity profile, clinical activity and pharmacokinetics (PK), allowing for approval of ch14.18/CHO for the use in phase-2 and -3 clinical trials [4].

Delivery of anti-GD_2_ Ab to pts is associated with the induction of pain [5,6,7], which is an on-target/off-tumor side effect. The mechanisms involved have been attributed to direct or indirect effects of the Ab on peripheral nerves [8]. It has been suggested that CDC is the primary mechanism of action responsible for the pain toxicity [9]. To overcome this obstacle, new anti-GD_2_ Abs with a mutated complement-binding region, such as hu14.18K322A, have been generated and used in clinical trials [9,10]. Indeed, strongly reduced complement activation was observed in pts treated with hu14.18K322A, resulting in some reduction of treatment toxicity [10]. In this report, the total opioid requirement in cycle 1 was reduced in pts treated with hu14.18K322A compared to pts treated with ch14.18 (1.57 vs. 2.41 mg/kg/d) [10]. However, hu14.18K322A treated pts still experienced pain requiring opioids, indicating that mechanisms other than complement activation may account for this on-target/off-tumor treatment toxicity.

Another approach to reduce pain toxicity is to decrease the delivery rate of the therapeutic Ab. In 2009, we initiated a single-center compassionate use program using a novel treatment schedule. Instead of STI over five days (5 × 20 mg/m^2^/d, 8 h infusion), 53 high-risk NB pts received the same cumulative dose given as a continuous long-term infusion (LTI) over 10 days (10 × 10 mg/m^2^/d) [11]. Pts received ch14.18/CHO in combination with IL-2 (6 × 10^6^ IU/m^2^/d, s.c.; d1–5 IL-2 alone, IL-2 in combination with ch14.18/CHO (10 × mg/m^2^/d); d8–12 and ch14.18/CHO alone; d13–18). The treatment protocol resulted in a reduced toxicity profile, with significantly reduced morphine usage and lower pain scores [11]. Importantly, strong induction of Ab-mediated effector functions was observed in this single-center cohort during the entire 6 month treatment period [12].

**Figure 1:**
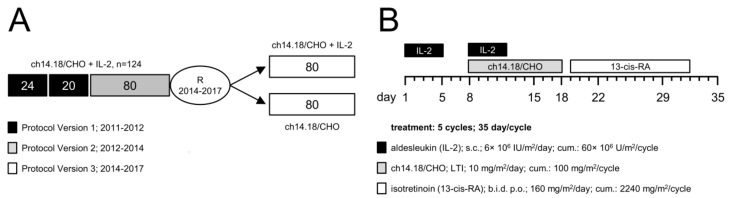
Schematic overview of the treatment schedule and the time line of the LTI study. (**A**) The LTI study (EudraCT-Number: 2009-018077-31) was planned as a single-arm study and amended in 2014 to address a randomized question. From 2011 to 2014, 124 pts were recruited in the single-arm phase, and 2 × 80 pts were recruited from 2014 to 2017 in the randomized phase. The single-arm phase consisted of a dose-finding (protocol version 1; 24 + 20 pts) and a dose confirmation cohort (protocol version 2; 80 pts), leading to a total of 124 pts in that part or the trial; (**B**) 122 of 124 enrolled NB pts received up to five treatment cycles (35 d/cycle) according to the following treatment protocol (2 pts progressed prior to first antibody application): IL-2 (aldesleukin; black horizontal bar) was given once a day for five days (s.c., d1–5, 6 × 10^6^ IU/m^2^/d), followed by a combined application of IL-2 once a day (s.c., d8–12, 6 × 10^6^ IU/m^2^/d) with a 10 days continuous infusion of ch14.18/CHO (i.v., d8–18, 10 mg/m^2^/d; grey horizontal bar). Starting on d19, treatment was continued with 13-cis-retinoic acid (isotretinoin; white horizontal bar) given twice a day (b.i.d) for the next 14 days (p.o., d19–32). Cumulative doses of IL-2, ch14.18/CHO and 13-cis-RA per cycle were 60 × 10^6^ IU/m^2^, 100 mg/m^2^ and 2240 mg/m^2^, respectively.

Based on these encouraging results, the SIOPEN group initiated a multi-center phase 2 clinical trial based on the LTI treatment schedule (EudraCT-Number: 2009-018077-31; LTI study). The trial was planned as a single-arm study and was amended in 2014 to address a randomized question (Figure 1). In the single-arm phase of the trial, 124 high-risk NB pts were enrolled for the treatment with 6 × 10^6^ IU/m^2^/d s.c. IL-2 (d1–5), followed by a combined application of IL-2 (d8–12) with LTI of ch14.18/CHO (10 × 10 mg/m^2^/d; d8–18).

Here, we report immunological results of the single-arm phase of the study focusing on the development of HACA, and the impact of this on ch14.18/CHO-dependent immunomodulation and pain toxicity. Validated pain scores and i.v. morphine usage was used to assess pain toxicity. For a comprehensive assessment of ch14.18/CHO concentrations in the pt circulation, a validated triple-ELISA method based on the anti-idiotype (anti-Id) Ab of ch14.18 ganglidiomab was applied allowing detection of a minimum concentration of 58 ng/mL ch14.18CHO (limit of detection) in pt serum [12,13]. To determine effector functions of ch14.18/CHO, such as patient-specific ADCC and CDC, we used two validated bioassays [14]. We also examined the frequency of human anti-chimeric antibody (HACA) responses in pt serum [15] and correlated these findings with ch14.18/CHO concentration-time curves and patient-specific effector functions ADCC and CDC as well as pain toxicity.

## 2. Results

### 2.1. Patient Selection and Treatment

Pts must be diagnosed with NB according to the INSS criteria [16]. Pts with high risk, primary refractory disease (≥2 lines of conventional treatment) or treated and responding relapse were eligible and enrolled in a European multi-center study (Figure 1).

In the single-arm phase of the trial, 124 pts were enrolled for the treatment with 10 days continuous LTI of 10 mg/m^2^/d ch14.18/CHO (d8–18) (cumulative dose of 100 mg/m^2^/cycle) combined with 6 × 10^6^ IU/m^2^/d s.c. IL-2 (d1–5; 8–12) (cumulative dose of 60 × 10^6^ IU/m^2^/cycle) and 160 mg/m^2^/d oral isotretinoin (d19–32) (cumulative dose of 2240 mg/m^2^/cycle) (Figure 1). A total number of five cycles (35 d/cycle) was planned. Since two pts had progressive disease prior to the treatment start, they were excluded from the analysis as they were not exposed to ch14.18/CHO. 88/122 pts received five immunotherapy cycles. 34/122 were treated with less than five cycles due to inflammatory side effects, infection or disease progression: four pts received four cycles, seven pts received three cycles, 14 pts received two cycles and nine pts received only one treatment cycle.

For pain assessment, a validated score system and i.v. morphine usage were used in participating centers and 122/124 pts were evaluable for these analyses.

### 2.2. Time Line of HACA Development in Treated Patients

**Figure 2:**
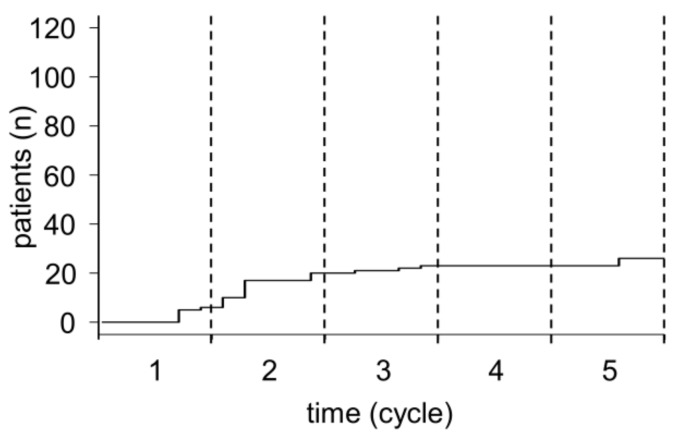
Time-line of HACA development during treatment with ch14.18/CHO. Serum samples collected from 122 pts treated with up to five cycles of ch14.18/CHO immunotherapy were analyzed in every cycle with a validated ELISA allowing for the detection of anti-ch14.18/CHO Abs (HACA). The cumulative incidence of HACA in treated pts during the treatment period is shown over time (black solid line). For a better overview, the end of the respective cycle is indicated by dashed vertical lines.

Based on the chimeric nature of ch14.18/CHO and our previous data showing development of HACA in 19% of NB pts treated with ch14.18/CHO [12], we evaluated HACA in the single-arm cohort of the LTI trial (122/124) using a validated ELISA [15]. Pts developing HACA above the limit of detection by ELISA at any time point during or after treatment were defined as HACA-positive pts. We detected HACA in 26/122 pts (21%), in line with observations made in the single center cohort [12]. In the majority of HACA-positive pts (17/26 pts, 65%), HACA were identified by the end of cycle 2 (Figure 2), but HACA were identified in 5/26 pts by the end of cycle 1 (d29) (Figure 2). Similar results were obtained in pts treated in the single center program where 7/10 (70%) of HACA pts were HACA positive at the end of cycle 2 (unpublished data). Surprisingly, in 3/122 pts we found HACA detectable for the first time on d29 of cycle 5 (late responders). Since these three pts developed HACA after the last Ab treatment, they were not included in the analysis for impact of HACA on ch14.18/CHO-dependent effector functions.

### 2.3. Serum Concentration of ch14.18/CHO

Ab serum concentrations were measured in every cycle at following time points: prior to Ab infusion (d1, d8), during Ab infusion (d15) and after end of Ab infusion (d18, d22 and d29) using a three step ELISA with a limit of detection at 58 ng/mL [12,13] as described in the “Materials and Methods” section.

**Figure 3:**
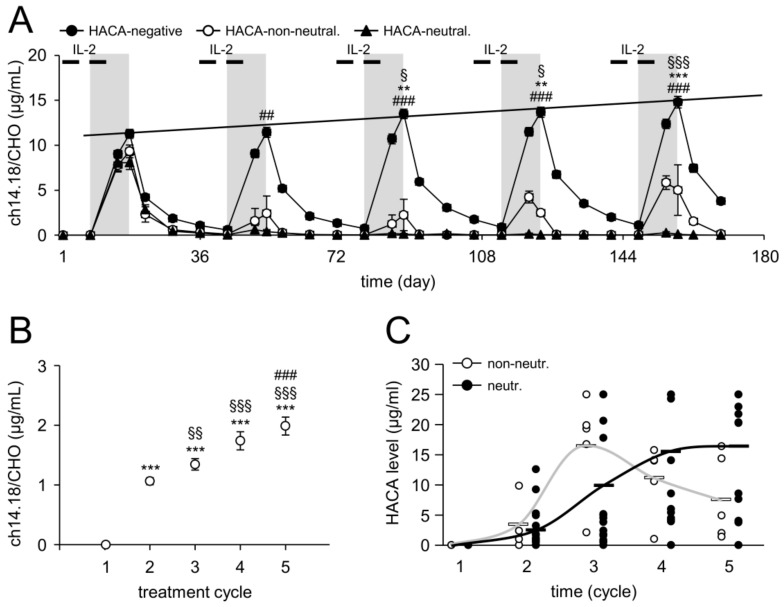
Serum levels of ch14.18/CHO and HACA in treated pts. (**A**) Samples collected from HACA-negative (99/122 pts, closed circles) and HACA-positive pts who developed non-neutralizing (5/23 pts, open circles) and neutralizing anti-ch14.18/CHO Ab (18/23 pts, closed triangles) were evaluated with the ch14.18/CHO-triple-ELISA strategy described in “Materials and Methods”. Ch14.18/CHO levels were analyzed prior to start, during and after the end of Ab infusion. The LTI of ch14.18/CHO is indicated by the gray field and IL-2 treatments by the black bars; (**B**) Baseline ch14.18/CHO concentrations in samples of HACA negative pts collected at d1 of every cycle. Data are shown as mean values ± SEM of experiments performed at least in triplicate. When error bars are not visible they are covered by the symbol. Solid line indicates the trend increase of maximum concentrations of ch14.18/CHO over time; (**C**) HACA serum levels of each HACA-positive pt of the non-neutralizing (5/23 pts, open circles) and neutralizing (18/23 pts, closed circles) cohort. White (non-neutralizing) and black solid horizontal bars (neutralizing) indicate mean values of the respective group. Grey and black solid lines indicate a trend of HACA levels during the entire treatment period in non-neutralizing and neutralizing pts, respectively. *t*-test or Mann-Whitney Rank Sum test. (**A**) ** *p* < 0.01 vs. d18, cycle 1; *** *p* < 0.001 vs. d18, cycle 1; ^§^ *p* < 0.05 vs. d18, cycle 2; ^§§§^ *p* < 0.001 vs. d18, cycle 2; ^##^ *p* < 0.01 vs. d18, cycle 2 of non-neutralizing pts; ^###^ *p* < 0.001 vs. d18 of the respective cycle of non-neutralizing pts; (**B**) *** *p* < 0.001 vs. d1, cycle 1; ^§§^ *p* < 0.01 vs. d1, cycle 2; ^§§§^ *p* < 0.001 vs. d1, cycle 2; ^###^ *p* < 0.001 vs. d1, cycle 3.

LTI of ch14.18/CHO in cycle 1 resulted in an increase of Ab serum concentrations during the infusion reaching the highest concentration at the end of infusion (11.26 ± 0.50 µg/mL; d18) (Figure 3A). Sequential increases in peak concentrations were observed in subsequent cycles (11.44 ± 0.56, 13.48 ± 0.52, 13.67 ± 0.56 and 14.79 ± 0.64 µg/mL for cycle 2, 3, 4 and 5, respectively). These data indicate accumulation of the therapeutic Ab over the entire treatment period (Figure 3A). We additionally analyzed ch14.18/CHO levels at the start of the following treatment cycles (d1). Compared to the baseline (d1, cycle 1), we revealed a highly significant increase of Ab levels from cycle to cycle (1.06 ± 0.06, 1.34 ± 0.09, 1.74 ± 0.15, and 1.99 ± 0.15 µg/mL for cycle 1, 2, 3, 4 and 5, respectively) (Figure 3B). Importantly, the increasing concentrations at these time points were found to be above the immunologically active Ab concentration of 1 µg/mL [3]. These data clearly show that the LTI of ch14.18/CHO results in active Ab concentrations for the entire duration of 6 month treatment period.

### 2.4. Impact of HACA on ch14.18/CHO Serum Concentration-Time Curves

First, we evaluated the impact of HACA on the Ab serum concentration-time curves in 23/122 HACA-positive pts. Based on the results of ch14.18/CHO-ELISA, we divided HACA-pts into two cohorts depending on whether the HACA response resulted in ch14.18/CHO serum levels above or below the immunologically active level of 1 µg/mL. Therefore, HACA pts showing a clearance (here defined as neutralizing) of ch14.18/CHO from the circulation (<1 µg/mL) were defined as neutralizing HACA pts and the second cohort with strongly reduced ch14.18/CHO (≥1 µg/mL, no clearance) at the end of infusion as non-neutralizing pts. With this definition, we identified 5/122 (4%) and 18/122 (15%) pts who developed non-neutralizing and neutralizing HACA responses, respectively. The ch14.18/CHO peak levels in neutralizing HACA pts were 0.40 ± 0.22, 0.13 ± 0.06, 0.05 ± 0.01, and 0.10 ± 0.06 µg/mL for cycle 2, 3, 4 and 5 (d18 of cycle; last day of Ab infusion), respectively, i.e., below the level of 1 µg/mL considered immunologically active (Figure 3A). The ch14.18/CHO concentration-time curves in pts with a non-neutralizing HACA response revealed significantly reduced ch14.18/CHO levels compared to HCAC-negative pts, however the ch14.18/CHO concentrations on the last day of ch14.18 LTI (d18) were above the immunologically active level of 1 µg/mL (d18 for cycle 2, 3, 4, and 5: 2.43 ± 1.93, 2.24 ± 1.74, 2.48 ± 0.36 and 5.01 ± 2.81 µg/mL, respectively) (Figure 3A).

To evaluate whether the HACA level determines development of non-neutralizing or neutralizing HACA responses, we compared them in neutralizing with non-neutralizing pts on d8 in each cycle (prior Ab infusion) (Figure 3C). There was no difference in HACA levels between neutralizing and non-neutralizing HACA pt cohorts. These data suggest that the type of HACA rather than the absolute level, determines whether HACA bind and/or neutralize the therapeutic Ab ch14.18/CHO. The kinetics of the development of non-neutralizing and neutralizing HACA differed; in pts with non-neutralizing HACA, HACA levels were highest after cycle 2, but then appeared to decrease in subsequent cycles, whereas a steady increase throughout the duration of treatment was observed in pts with neutralizing HACA.

### 2.5. Antibody-Dependent Cell-Mediated Cytotoxicity

**Figure 4:**
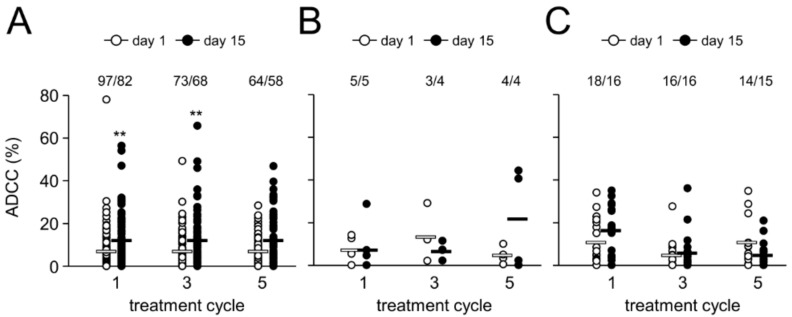
Ch14.18/CHO-mediated ADCC and impact of HACA response. Induction of GD_2_-specific ch14.18/CHO-mediated ADCC in HACA-negative (**A**) and HACA-positive pts (non-neutralizing (**B**) and neutralizing pts (**C**)) treated with the LTI regimen was analyzed in cycles 1, 3 and 5 on d15 (closed circles) and compared to the baseline cytotoxicity of the respective cycle (d1; open circles). ADCC was evaluated against the GD_2_-positive NB cells LAN-1 as described in “Materials and Methods”. The circles represent pts evaluable for the analysis (number of pts are shown above the respective groups). Experiments were performed in six replicates. White (non-neutralizing) and black solid horizontal bars (neutralizing) indicate mean values of the respective group. *t*-test or Mann-Whitney Rank Sum test. ** *p* < 0.01 vs. d1 of the respective cycle.

Since ADCC has been reported to be a major mechanism of action of therapeutic Ab [17], we analyzed ch14.18/CHO-dependent effector cell-mediated cytotoxicity against LAN-1 NB cells (ADCC) using a validated cytotoxicity assay [14]. We observed a two-fold increase of patient-specific ADCC on d15 (i.e., d8 of Ab infusion) in cycles 1, 3 and 5 compared to d1 in the respective cycles (Figure 4). This increase was highly significant in cycle 1 and 3, but not in cycle 5, probably due to a low number of samples available for the analysis.

### 2.6. Effect of HACA on Antibody-Dependent Cell-Mediated Cytotoxicity

We then evaluated the effect of a HACA response on ADCC levels in neutralizing and non-neutralizing HACA responders and compared these results with those from HACA negative pts.

In line with our results obtained with ch14.18/CHO concentration-time curves, increased ADCC levels were observed in cycle 1 in the neutralizing pt group (Figure 4C), in keeping with the fact that HACA development was only observed after cycle 2 in the majority of pts. However, the difference was not statistically different, probably due to small number of available pts. Interestingly, HACA non-neutralizing pts did not show any change of ADCC even in cycle 1 (Figure 4B). Consequently, compared to HACA-negative pts, ADCC in cycles 3 and 5 in HACA-positive pts was observed to be similar to the baseline (Figure 4B,C). In the majority of pts with a neutralizing HACA-response we observed a complete abrogation of ADCC on d15 (i.e., d8 of ch14.18/CHO LTI) showing similar cytotoxicity levels as prior to Ab infusion (7 ± 1%) (mean of 5 ± 2% vs. 6 ± 1% and 11 ± 3% vs. 5 ± 2% for d1 and 15 in cycle 3 and 5, respectively) (Figure 4C). However, in cycle 3 and 5 on d15 we observed ADCC levels above baseline (11% ± 2%) in three and two neutralizing pts, respectively, indicating active cellular cytotoxicity despite HACA-dependent neutralizing of ch14.18/CHO (Figure 4C). Although we observed in cycle 3 on d15 in HACA non-neutralizing pts a complete abrogation of ADCC (7 ± 2%), ADCC found in cycle 5 on d15 (22 ± 12%) was higher compared with the baseline value (8 ± 3%) indicating induction of cellular cytotoxicity despite strong reduction of ch14.18/CHO in pts circulation (Figure 4B). These results emphasize HACA-dependent impact on cell-mediated effector functions of the therapeutic Ab with the exception of few HACA-positive pts showing active cellular cytotoxicity despite neutralization of ch14.18/CHO.

### 2.7. Complement-Dependent Cytotoxicity

Complement activation is an important effector function of mAbs and has also been postulated to be a key mechanism involved in pain toxicity associated with anti-GD_2_ Ab [9]. Since LTI with ch14.18/CHO was reported to reduce pain toxicity [18], we addressed the question whether the complement-mediated anti-tumor cytotoxicity is affected by the slower infusion rate in LTI pts. 

**Figure 5:**
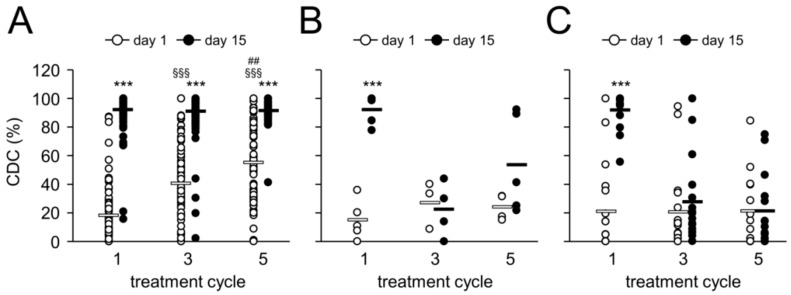
Ch14.18/CHO-mediated CDC and impact of HACA response. Induction of GD_2_-specific ch14.18/CHO-mediated CDC in HACA-negative (**A**) and HACA-positive pts who developed non-neutralizing (**B**) and neutralizing anti-ch14.18/CHO Ab (**C**) was analyzed. CDC was tested in cycles 1, 3 and 5 on d8 after the start of Ab infusion (i.e., d15; closed circles) and compared to the baseline CDC of the respective cycle (d1; open circles) using the cytotoxicity assay as described in “Materials and Methods”. The circles represent pts evaluable for the analysis. Experiments were performed in six replicates. White (non-neutralizing) and black solid horizontal bars (neutralizing) indicate mean values of the respective group. *t*-test or Mann-Whitney Rank Sum test. *** *p* < 0.001 vs. d1 of the respective cycle; ^§§§^ *p* < 0.001 vs. d1, cycle 1; ^##^ *p* < 0.001 vs. d1, cycle 3.

We analyzed CDC at the same time points as ADCC, i.e., on d1 and d15 (i.e., d8 of Ab infusion) in cycles 1, 3 and 5 using the validated cytotoxicity assay as previously described [14]. In line with our ADCC data, we found a strong and highly significant induction of CDC on d15 compared to the baseline in all cycles analyzed in HACA negative pts (Figure 5A). The observed CDC level on d15 was almost 100% indicating the highest possible complement activation by LTI of ch14.18/CHO. Importantly, in accordance to the observed accumulation of ch14.18/CHO in the subsequent cycles, even prior to start of Ab infusion we found a highly significant increase of CDC prior to the Ab infusion at these time points in cycles 3 and 5 compared to the baseline (d1 of cycle 1). These data show a strong complement activation not only during the antibody delivery but also between the cycles allowing anti-tumor response during the entire treatment period of six months. 

Interestingly, despite of a complement activation leading to 100% cytotoxicity on d15 (i.e., d8 of Ab infusion), pain toxicity level was low (below 2 out of 10 score units) allowing morphine-free delivery of ch14.18/CHO in the majority of pts. These results suggest that complement activation is not the only mechanism responsible for the pain side effect.

We also compared CDC levels in neutralizing with non-neutralizing HACA pts (Figure 5B,C). Again, in cycle 1 there was no difference in CDC levels between the both pt cohorts (92 ± 3% and 92 ± 5% for neutralizing and non-neutralizing HACA, respectively, *p* > 0.1) as HACA develops after cycle 2 in the majority of pts. In contrast, in pts who developed neutralizing HACA, CDC was completely abrogated in most pts in subsequent cycles showing levels not different from the baseline values (Figure 5C). However, similar to ADCC, we observed increased CDC in few pts with a neutralizing HACA response, indicating active complement cytotoxicity despite HACA-dependent neutralization of ch14.18/CHO (Figure 5C). Analysis of CDC on d15 in cycle 3 and 5 revealed in non-neutralizing HACA significantly reduced cytotoxicity compared to HACA-negative pts (*p* < 0.001). Interestingly, CDC levels in HACA-non-neutralizing pts in cycle 5 were still higher compared to the baseline level and HACA-neutralizing pts (54 ± 15% vs. 15 ± 6% for d15 of cycle 5 and baseline, respectively) (Figure 5B), however, the differences were not statistically significant probably due to a low number of pts. These data show a clear correlation between ch14.18/CHO serum concentrations and CDC levels and are in line with our ch14.18-ELISA- and ADCC results again with few exemptions where CDC was observed despite neutralization of ch14.18/CHO.

### 2.8. Treatment Toxicity

**Figure 6:**
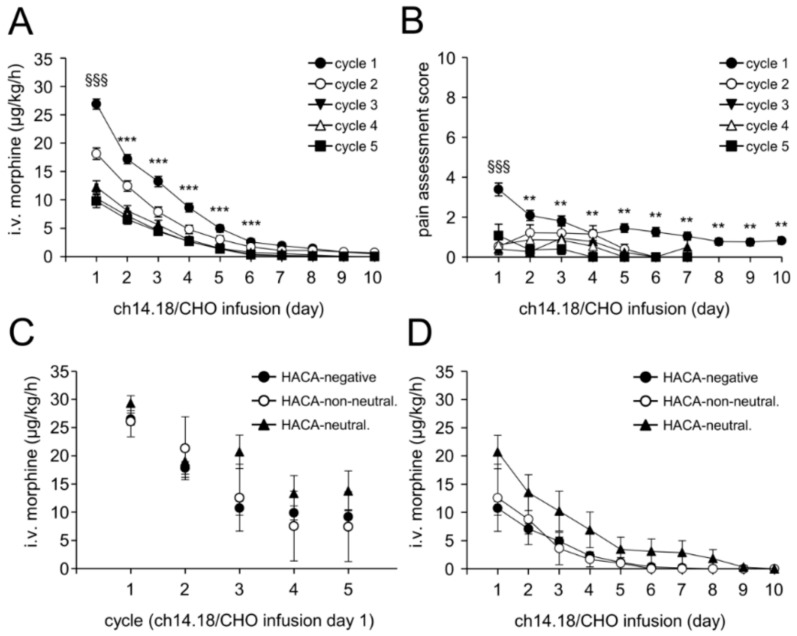
Intravenous morphine usage and pain assessment. Ch14.18/CHO-dependent pain toxicity was evaluated by systematic assessments of i.v. morphine usage (**A**,**C**,**D**) and pain scores (**B**) in every treatment cycle during antibody administration (LTI, 10 days) as described in “Materials and Methods”. (**A**) Usage of i.v. morphine in μg/kg/d was determined daily per pt and cycle and presented as mean ± SEM. When error bars are not visible, they are covered by the symbol; (**B**) Pain assessment scores were determined daily per pts and cycle using two validated age-adapted pain score systems. Values represent mean maximum pain scores ± SEM; (**C**) Comparison of i.v. morphine usage in every cycle on d8 (i.e., d1 of ch14.18/CHO infusion) in HACA-negative pts (closed circles) and pts with neutralizing (closed triangles) and non-neutralizing HACA (open circles); (**D**) Daily i.v. morphine usage in cycle 3 in HACA-negative pts (closed circles) and pts with neutralizing (closed triangles) and non-neutralizing HACA (open circles). Data represent mean ± SEM. When error bars are not visible they are covered by the symbol. *t*-test or Mann-Whitney Rank Sum test. (**A**) *** *p* < 0.001 vs. d1, cycle 1; ^§§§^ *p* < 0.001 vs. d1, cycle 2, 3, 4 and 5; (**B**) ** *p* < 0.01 vs. d1, cycle 1; ^§§§^ *p* < 0.001 vs. d1, cycle 2, 3, 4 and 5.

To determine the treatment toxicity, i.v. morphine usage and pain intensity were evaluated in each treatment cycle and throughout the entire treatment period. Analysis of i.v. morphine showed a steady decrease in requirement even within the first cycle (Figure 6A) from 0.66 ± 0.02 mg/kg on d8 (i.e., d1 of LTI) to 0.03 ± 0.01 mg on d15 (i.e., d8 of LTI). 

In line with this, we observed a further decrease also in the subsequent cycles. Importantly, comparison of i.v. morphine usage on d8 (i.e., d1 of ch14.18/CHO application) between the treatment cycles revealed a steady decrease of morphine usage in the following cycles: 0.48 ± 0.02, 0.37 ± 0.03, 0.33 ± 0.03 and 0.33 ± 0.03 mg/kg for cycle 2, 3, 4 and 5, respectively. These data clearly show a decrease of the morphine usage not only during the cycle but also from cycle to cycle throughout the entire immunotherapy. Already on d6 of ch14.18/CHO LTI, morphine-free treatment was possible in 74%, 88%, 93%, 96% and 94% pts in cycle 1, 2, 3, 4, and 5, respectively.

We then analyzed pain levels in pts using our validated assessment (pain score scale: 1–10 units) and observed a very low pain toxicity level which was in the range of three score units (Figure 6B). Comparison to the subsequent cycles showed a further decrease of pain levels. These data show an improved treatment tolerance and a decreased toxicity of the LTI therapy. Importantly, these parameters were further improved in the subsequent cycles.

### 2.9. Impact of HACA on Treatment Toxicity

Since HACA affected ch14.18/CHO concentration-time curves showing strongly reduced Ab levels and in particular also reduced CDC levels in HACA-positive pts, we addressed the question, whether HACA has any impact on pain toxicity. For this, i.v. morphine usage of HACA-negative pts was compared to that of HACA neutralizing and -non-neutralizing pts. We compared the i.v. morphine usage in every cycle on d8 (i.e., d1 of ch14.18/CHO infusion) in these three pt cohorts (Figure 6C) and did not observe any significant differences between them.

This finding was confirmed by the analysis of the daily i.v. morphine usage in cycle 3, which was selected as most HACA-positive pts developed HACA before cycle 3. There was no correlation between HACA and i.v. morphine usage (Figure 6D) or pain intensity (data not shown).

Interestingly, pts with neutralizing HACA showed a tendency towards a higher i.v. morphine usage compared to HACA-negative and HACA-non-neutralizing pts. However, these differences were statistically not significant (Figure 6D). In summary, HACA response abrogated CDC but did not affect pain intensity supporting that there are other mechanisms responsible for the pain toxicity observed with this treatment regimen.

### 2.10. Evaluation of an Anti-Anti-Id Response

In HACA-positive pts we investigated the induction of an adaptive immune response according to the idiotype network theory. According to this, ch14.18/CHO application (Ab1) can result in HACA induction (Ab2) representing the second step in the formation of the anti-Id network. Ab2 can induce production of anti-anti-Id Ab (Ab3) which binds to the nominal antigen of Ab1, i.e., GD_2_. Therefore, we used a GD_2_-based solid phase ELISA to analyze Ab3 in serum samples of HACA-positive pts as previously described [19]. To avoid any cross-reaction between GD_2_ and ch14.18/CHO, only HACA-positive samples lacking ch14.18/CHO were analyzed. Compared to the respective baseline samples (d1, cycle 1), we did not observe any detectable anti-anti-Id in any evaluable HACA-positive pt (data not shown) with this method.

## 3. Discussion

GD_2_ directed immunotherapies have improved survival in high-risk NB pts [2]. However, the treatment is associated with induction of neuropathic pain, which is managed by intensive co-medication including administration of i.v. morphine. To reduce pain toxicity, we developed a new delivery method administering the chimeric anti-GD_2_ Ab ch14.18/CHO as a continuous 10 days infusion (LTI) of 100 mg/m^2^ Ab per cycle. In our compassionate use program, this slow continuous delivery showed marked improvement in treatment tolerance [11,18]. At the same time, we reported effective induction of Ab dependent anti-NB immune responses mediated by effector cells and serum proteins of treated pts [12], and we identified the HACA response rate of 19% in this cohort [15].

The SIOPEN LTI study was initiated to validate some of the major findings of the compassionate use program. Here, we report the frequency and the impact of HACA in 124 NB pts enrolled to version 2 of the clinical trial protocol (single-arm part of the trial) on the effector functions of the therapeutic Ab ch14.18/CHO and on pain.

Therapies with murine or mouse/human chimeric Ab directed against GD_2_-positive NB have been shown to induce an Ab response against the therapeutic Ab. The first generation of Ab-based therapies relied on entirely murine anti-GD_2_ Ab, such as 14G2a. Almost all pts treated with 14G2a showed a human anti-mouse Ab (HAMA) response (88–100%) [20,21,22] leading to the formation of immune complexes on repeated administrations, which resulted in elimination of the therapeutic Ab from the patient circulation. It is still not clear, if such immune responses are a disadvantage with respect to survival of treated pts [23]. Nevertheless, the mouse/human version, ch14.18 was developed, consisting of murine variable and human constant regions. In initial studies, 70% (7/10) pts treated with ch14.18 developed anti-chimeric Ab responses (HACA) response [7] indicating that ch14.18 had a lower immunogenicity compared to completely murine anti-GD_2_ Ab. Recently, we described HACA in 19% (10/53) of treated pts in the single-center program [12] and confirm the HACA rate at 21% (26/122) in the single-arm phase of the LTI study (Figure 2) underlining the lower immunogenicity of the chimeric anti-GD_2_ Ab. The role of the co-treatment with IL-2 for HACA development is not clear up to date, and will be addressed in the randomized phase of the LTI study. Induction of HACA against therapeutic chimeric Ab has been also reported in pts treated with chimeric anti-CD20 Ab rituximab with HACA rates ranging from 10 to 40% [24,25,26].

Importantly, the HACA response inversely correlates with the ch14.18/CHO serum level as well as with ADCC and CDC (Figure 3, Figure 4 and Figure 5). Prior to the HACA development, we observed a strong increase of ch14.18/CHO levels and induction of ADCC and CDC in all pts analyzed (Figure 3, Figure 4 and Figure 5). In contrast, only pts who developed HACA responses showed either a clearance of ch14.18/CHO from the circulation (Ab levels <1 µg/mL; neutralizing) or strongly reduced ch14.18/CHO levels (here defined as non-neutralizing) as well as reduced or abrogated ADCC and CDC in the majority of pts, underlining the reliability of our HACA detection method giving meaningful results (Figure 3A). Interestingly, we did not find higher HACA levels in pts with a neutralizing HACA response compared to pts with a non-neutralizing HACA response. Surprisingly, we observed in cycle 3 even higher HACA levels in non-neutralizing pts, however, in cycle 4 and 5 higher HACA levels in pts with neutralizing HACA were detected (Figure 3C). 

These data suggest that the type of HACA rather than the absolute level define whether HACA reduce or neutralize the therapeutic Ab ch14.18/CHO. Binding affinity and binding site are important parameters that influence the effect of HACA on the therapeutic Ab ch14.18/CHO. However, the reasons for the different ch14.18/CHO kinetics observed in neutralizing and non-neutralizing HACA pts need further clarification.

In the context of the described HACA dependent effects, the important question arises, whether HACA-positive pts should be treated differently. Since HACA impact on survival is not clear to date and the fact that HACA could induce the development of anti-anti-Id antibodies and thereby lead to an active immune response against GD_2_, HACA may also prolong survival. Therefore, we cannot recommend any change in the treatment protocol based on these results as the overall and event free survival data are not mature enough to correlate HACA response with outcome, which will be subject to future reports. Moreover, we showed that LTI of ch14.18/CHO resulted not only in effective immune modulation but also allowed reduction of pain toxicity similar to the observations made in the single-center program [11,18].

The mechanisms of pain induction after anti-GD_2_ Ab infusion are still not fully understood. It has been shown that anti-GD_2_ Ab have access to the peripheral nervous system following systemic dosing [27]. Furthermore, studies of 14G2a showed its binding to myelin in peripheral nerves [27] as well as to dorsal root ganglion cells [28] suggesting that this is the most likely cause of the side effect.

Due to the ability of anti-GD_2_ Ab (IgG2a, IgG1) to fix complement [9], the activation of the classical pathway of the complement system has been suggested as one of the mechanisms of pain induction. Indeed, spinal administration of soluble complement receptor 1 which blocks the formation of complement components C3a and C5a as well as the formation of the membrane attack complex reduced pain in rat models [9,29]. Based on these and other observations, the humanized anti-GD_2_ Ab hu14.18K322A with a single point mutation (K322A) abrogating complement activation was developed and assessed in a Phase 1 study given as STI to determine the safety profile. Indeed, pts treated with hu14.18K322A showed lower opioid usage compared to those who received ch14.18 [10,30]. However, despite the deficiency of complement activation by hu14.18K322A, pain was observed in treated pts requiring opioid co-medication which suggests that there are other mechanisms of pain induction than CDC.

This contention is supported by our finding in pts with abrogated CDC as a result of their HACA response experienced the same level of pain as HACA negative pts with fully established CDC levels (Figure 6).

Recently, cytokines, such as IL-1 beta, IL-6, TNF and IL-17, were shown to be involved in pain development [31,32]. Besides their classical role some cytokines can directly activate nociceptors and induce pain hypersensitivity [31]. Several studies showed that cytokine blockade was effective in reducing pain. For example, intrathecal injection of both IL-1-beta and TNF-alpha antagonists alleviated pain induced by spinal injury [33] and IL-6 neutralizing Ab reduced mechanical allodynia and down-regulated the expression of IL-1 beta and TNF-alpha within the central nervous system [34]. Similar effects could be observed after treatment with TNF-alpha-, IL-1 beta- and IL-6 inhibitors [31]. Studies of IL-17 involvement in pain induction have shown that knock-out of this cytokine in mice resulted in reduction of pain hypersensitivity [35]. It is not clear to what extent cytokines are involved in ch14.18/CHO mediated induction of neuropathic pain. This question can be addressed in randomized trials.

In summary, we demonstrate that LTI of ch14.18/CHO in combination with IL-2 results in a strong activation of Ab effector functions. We observed a HACA response rate of 21%, which abrogated CDC but did not affect pain suggesting other mechanisms of ch14.18/CHO mediated pain induction than CDC.

## 4. Materials and Methods

### 4.1. Ethic Statement

All procedures involving human participants were in accordance with the ethical standards of the institutional and national research committees and competent authorities and with the 1964 Helsinki declaration and its later amendments or comparable ethical standards. Treatment conducted under this multi-center single-arm phase 2 clinical trial (EudraCT-Number: 2009-018077-31; LTI study protocol version 1 (24 + 20 pts, 2011–2012), version 2 (80 pts, 2012–2014) (Figure 1). and analysis protocols were approved by the ethical committees of participating centers (University Children’s Hospital, Greifswald, Germany (ethical code: MV 05/11); St. Anna Children’s Hospital, Vienna, Austria (ethical code: 693/2010); Great Ormond Street, London, UK (ethical code: 012010); Children’s Hospital, Birmingham, UK (ethical code: 12/WM/0079); University Hospital, Bristol, UK (ethical code: 12/WM/0079); Leeds Teaching Hospital, Leeds, UK (ethical code: 12/WM/0079); Alder Hey Children’s, Liverpool, UK (ethical code: 12/WM/0079); the Newcastle upon Tyne Hospitals, Newcastle, UK (ethical code: 12/WM/0079); UCLH, London, UK (ethical code: 12/WM/0079); Our Lady’s Children’s Hospital Crumlin, Dublin, Ireland (ethical code: 012010); University Children’s Hospital, Jena, Germany (ethical code: 3569-09/12); University Children’s Hospital, Frankfurt, Germany (ethical code: 330/12); Istituto Nazionale dei Tumori, Milan, Italy (012010); Gaslini Children’s Hospital, Genova, Italy (012010); Hospital Universitario La Fe, Valencia, Spain (ethical code: 012010); Institut Curie, Paris, France (ethical code: IGR2010/1712); Institut Gustave Roussy, Villejuif, France (ethical code: IGR2010/1712); Schneider Children’s Medical Centre of Israel; Petach Tikvah, Israel (ethical code: FWA00009930). Informed consent was obtained from all individual participants or their parents or legal guardians.

### 4.2. Patients

124 NB pts enrolled in the single-arm phase of the trial were treated with ch14.18/CHO given as LTI in combination with IL-2 given subcutaneously (Figure 1). Only pts with biopsy-proven high-risk NB were treated. Pts after first-line therapy had to have evaluable disease. Following second-line chemotherapy, pts were allowed to be treated without evidence of disease; previous treatment had to be discontinued three weeks prior to the start of ch14.18/CHO. Treatment with isotretinoin, growth factor or other immunomodulatory therapy needed to be completed at least seven days before treatment start. A performance score above 70% and a life expectancy of at least 12 weeks were required for inclusion. Following treatment protocol was used (Figure 1): cytokine IL-2 was given for five days (s.c., d1–5, 6 × 10^6^ IU/m^2^/d), followed by a combined application of IL-2 (s.c., d8–12, 6 × 10^6^ IU/m^2^/d) with ch14.18/CHO (i.v., d8–18, 10 mg/m^2^/d) and 13-cis-retinoic acid (p.o., d19–32). At treatment start, the performance scores were ≥90 (Lansky or Karnofsky).

### 4.3. Sampling

To analyze ch14.18/CHO and HACA in pt circulation, serum samples were used. For ch14.18/CHO analysis, samples were collected in every treatment cycle prior to Ab infusion (d1 and d8), during Ab infusion (d15), at the end of Ab infusion (d18) and after Ab infusion (d22 and d29). For HACA analysis, serum collected in every treatment cycle between d8 (prior to Ab infusion) and d29 (11 days after end of Ab infusion) was used. Serum samples were prepared from clotted blood samples by centrifugation for 10 min at 1700× *g*, RT and stored in aliquots at −80 °C. For analysis of ADCC and CDC, pt sodium-heparin blood and serum were collected in cycle 1, 3 and 5 prior to start of immunotherapy (d1) and on d8 of ch14.18/CHO infusion (d15 of cycle).

### 4.4. Analysis of Treatment Toxicity

To evaluate the toxicity of this treatment regimen, we assessed i.v. morphine usage and pain levels in every treatment cycle after start of Ab infusion (10 days: d1–10 corresponding to d8–18 of treatment cycle). Pain levels were daily evaluated by the medical team using validated age-adapted pain scores (KUSS, MOPS and Ramsay scores) [36,37,38,39]. Maximum pain score of each day was used for statistical analysis. Additionally, the usage of i.v. morphine needed during the Ab application was comprehensively assessed.

### 4.5. Analysis of ch14.18/CHO Levels in Patient Serum

Validated detection of ch14.18/CHO in pt samples was performed using the triple-ELISA strategy (limit of detection in serum samples: 58 ng/mL ch14.18/CHO) as previously described [12,14]. The anti-idiotype Ab ganglidiomab [19] was used as capture Ab. Briefly, pt serum samples were first analyzed using the “low sensitivity” ELISA with a detection range of 3.0–25 µg/mL ch14.18/CHO. Then, samples containing lower ch14.18/CHO levels than 3 µg/mL were subjected to reanalysis with the “intermediate sensitivity” ELISA (detection range: 0.5–3.1 µg/mL). Finally, samples with ch14.18/CHO concentrations below 0.5 µg/mL were reanalyzed with the “high sensitivity” ELISA with detection range of 0.058–1.0 µg/mL.

### 4.6. Evaluation of Human Anti-Chimeric Ab Response

To analyze HACA development in pts treated with ch14.18/CHO, a validated ELISA allowing specific detection of anti-ch14.18/CHO Ab in pt serum was performed as previously described [15].

### 4.7. Analysis of ADCC and CDC

To evaluate ADCC and CDC effector functions induced by ch14.18/CHO, patient-specific effector cells and serum were analyzed using a cytotoxicity assay based on a calcein-acetoxymethyl ester (AM) as previously described [14]. Briefly, pt serum for CDC (12.5% final concentration) as well as leukocytes and heat-inactivated serum for ADCC (effector-to-target cell ratio: 40:1) were incubated with 5000 calcein-AM-labeled human GD_2_-positive NB cells LAN-1 for 4 h. Heat-inactivated serum without leukocytes served as negative control for CDC analysis. Cytotoxic activity was determined by measuring of the calcein fluorescence in the supernatants at 495 nm excitation and 515 nm emission wavelengths. ADCC was calculated according to the formula: (experimental release − spontaneous release (target cells only))/(maximum release (target cells disrupted using an ultrasonic homogenizer) − spontaneous release) × 100%. For CDC, the following formula was used: (experimental release − negative control release)/(maximum release − negative control release) × 100%. All samples were analyzed in at least six replicates.

### 4.8. Statistics

Statistical analysis was performed using Sigma Plot software (Version 13.0, Systat Software GmbH, D-40699 Erkrath, Germany). After testing for normality and equal variance across groups, differences between groups were assessed using either the *t*-test or Mann-Whitney rank sum test or Wilcoxon signed-rank test. A *p* value of <0.05 was considered significant, <0.01 very significant and <0.001 highly significant. All data are presented as mean ± SD (standard deviation) or mean ± SEM (standard error of the mean).

## 5. Conclusions

In the present study, we showed that prolongation of the application time of the anti-GD_2_ Ab ch14.18/CHO by the long-term infusion resulted not only in effective immune modulation (ADCC and CDC) but also allowed reduction of pain toxicity, which is known to be a limiting factor after short-term infusions of anti-GD_2_ Ab. In previous reports, the mechanism of pain induction was related to the activation of the complement system. 

In our clinical trial, we observed the development of HACA in 21% of pts which resulted in strong reduction of ch14.18/CHO levels and abrogation of CDC and ADCC. Importantly, HACA-positive pts, who had completely abrogated CDC did not show any change of pain toxicity compared to the HACA-negative pts who had strongly activated CDC indicating that there are mechanisms other than CDC involved in ch14.18/CHO induced pain induction.
